# A novel combination of fipronil and permethrin (Frontline Tri-Act®/Frontect®) reduces risk of transmission of *Babesia canis* by *Dermacentor reticulatus* and of *Ehrlichia canis* by *Rhipicephalus sanguineus* ticks to dogs

**DOI:** 10.1186/s13071-015-1207-5

**Published:** 2015-11-19

**Authors:** Frans Jongejan, Christa de Vos, Josephus J. Fourie, Frederic Beugnet

**Affiliations:** Utrecht Centre for Tick-borne Diseases (UCTD), FAO Reference Centre for Ticks and Tick-borne Diseases, Faculty of Veterinary Medicine, Utrecht University, Yalelaan 1, 3584 CL Utrecht, The Netherlands; Department of Veterinary Tropical Diseases, Faculty of Veterinary Science, University of Pretoria, Private Bag X04, Onderstepoort, 0110 South Africa; ClinVet International (Pty) Ltd, P.O. Box 11186, Universitas, Bloemfontein, 9321 Republic of South Africa; Merial S.A.S., 29 Av Tony Garnier, 69007 Lyon, France

**Keywords:** *Babesia canis*, *Ehrlichia canis*, *Dermacentor reticulatus*, *Rhipicephalus sanguineus*, Fipronil, Permethrin, Transmission blocking studies

## Abstract

**Background:**

The ability of Frontline Tri-Act®/Frontect®, a topical ectoparasiticide containing fipronil and permethrin for dogs, to prevent the transmission of *Babesia canis* as well as *Ehrlichia canis* was evaluated by infesting dogs with infected vector ticks.

**Methods:**

For the *Babesia canis* study, 16 dogs were randomly allocated to two groups. Eight dogs were treated on day 0 with a topical spot-on formulation containing 6.76 % *w/v* fipronil plus 50.48 % *w/v* permethrin and eight dogs served as the untreated control group. *Dermacentor reticulatus* ticks, with a *B. canis* infection rate ranging between 2 and 10 %, were placed onto dogs on days 7, 14, 21 and 28. *In situ* tick counts were performed on Days 9, 16 and 23. Ticks were counted and removed on Day 30. Infection of the dogs with *B. canis* was monitored by rectal temperature readings, clinical examinations and blood smears as well as PCR and IFA (indirect fluorescent antibody assay).

For the *Ehrlichia canis* study, another 16 dogs were allocated to two groups. Eight dogs were treated with the fipronil and permethrin combination on days 0 and 28 and eight dogs served as untreated controls. *Rhipicephalus sanguineus* ticks*,* carrying an infection rate of 13 % for *E. canis,* were released in the sleeping kennels of the dogs on days 7, 14, 21, 28, 35, 42, 49 and 56. Ticks were counted *in situ* on the dogs on a weekly basis. All ticks were removed and counted on the final assessment day 58. Infection of the dogs with *E. canis* was monitored by rectal temperature, clinical examinations, and testing of blood samples by PCR, IFA and platelet counts.

**Results:**

*B. canis* was transmitted by *D. reticulatus* ticks to all eight untreated control dogs and to one treated dog, which was confirmed by blood smears, PCR and IFA. *E.canis* was transmitted by *R. sanguineus* ticks to all eight untreated control dogs. Two of the dogs in the treated group were found positive based on PCR and/or IFA.

**Conclusions:**

Frontline Tri-Act®/Frontect® significantly lowered the risk for dogs to acquire a *B. canis* infection by 87.5 % over a challenge period of 28 days. The risk for dogs to acquire *E. canis* was reduced by 75 % over a period of 56 days.

## Background

Hard ticks (Acari: Ixodidae) infest dogs all over the world, causing direct damage due to high tick burdens. Their main importance, however, is related to their capacity to transmit a wide range of pathogenic micro-organisms [1, 2]. As a result, there is a continuous need to develop novel and/or combine existing tick control compounds for sustained tick control on companion animals, in particular dogs. Improved acaricidal formulations and combinations that are easy to administer, long acting, fast killing, and reduce infection by tick-borne diseases, e.g. babesiosis and ehrlichiosis, do provide value to veterinarians and their clients.

Canine babesiosis is caused by a number of different protozoan species of the genus *Babesia*, which vary in virulence and have expanded their distribution in recent years in particular in Europe [3].

Here the focus is on *Babesia canis*, where the occurrence largely coincides with the distribution of the ornate dog tick, *Dermacentor reticulatus* (Fabricius, 1794), a Palearctic species with a highly focal distribution pattern [4]. This tick occurs in foci in south-western England in the west all the way into Central Asia reaching the Yenisei river basin in Siberia in the east [5].

The second focus is on *Ehrlichia canis,* the causative agent of canine monocytic ehrlichiosis, which is transmitted by the brown dog tick, *Rhipicephalus sanguineus* (Latreille, 1806), and found worldwide anywhere between 50° N and 30° S [6].

Guidelines for conducting veterinary clinical studies have traditionally focussed on demonstrating acaricidal efficacy against ticks [7]. However, because of the importance of ticks as vectors of pathogens causing diseases in dogs and humans, there is an increasing demand for control methods that do not only kill ticks, but are also able to reduce the transmission of disease. Fipronil spotted onto dogs was shown to prevent infection with *E. canis* transmitted by *R. sanguineus* in Senegal [8]. Furthermore, application of amitraz-impregnated collars onto dogs in South Africa prevented infections with *Babesia rossi* transmitted by *Haemaphysalis elliptica* ticks [9]. In this particular study eight of 30 control dogs (26.6 %) became infected over a 6-month period compared to none of the 20 treated dogs. Field trials, however, depend on locally occurring challenge pressure, which often results in unpredictable numbers of untreated control animals contracting the tick-transmitted disease. Over the past couple of years, laboratory models that allow for a much more standardised evaluation of the transmission blocking ability of acaricidal compounds have been developed both for *B. canis* [10] as well as for *E. canis* [11]. As a result, the WAAVP recognised this development and included in their recent guidelines that specific claims regarding the prevention or reduction of tick-borne pathogen transmission are now possible [12]. However, specific recommendations regarding the design of pathogen blocking studies have not yet been included in any of the regulatory guidelines [13].

Both transmission blocking models were initially used to determine the level of transmission blocking of *B. canis-*infected *Dermacentor* ticks and *E. canis-*infected *Rhipicephalus* ticks applied onto dogs treated with a combination of fipronil, amitraz and (s)-methoprene (CERTIFECT^TM^) [10, 11]. Two additional studies were conducted with the *E. canis* blocking model; one study addressed the preventive capacity of a topical combination of imidacloprid and permethrin and the second study focussed on an imidacloprid and flumethrin collar for dogs [14, 15]. In addition to the topically active compounds [10] and slow release collar matrices [16], both recently discovered novel systemic compounds, afoxolaner [17] and fluralaner [18]) were also tested for their capacity to block transmission of *Babesia* [19, 20].

Recently, a combination of fipronil and permethrin (Frontline Tri-Act®/Frontect®) was tested for its acaricidal efficacy against *D. reticulatus* ticks [21] and also against *R. sanguineus* ticks [22]. Fipronil is a phenylpyrazole, which has been widely used as an acaricide/insecticide [23]. Permethrin, is a synthetic pyrethroid with a residual acaricidal activity as well as repellency effect sensu lato against ticks.

The studies reported in this paper assessed whether this combination is capable of preventing the transmission of *Babesia* as well as *Ehrlichia* using established transmission blocking models.

## Methods

### Study design and treatments

Both studies were conducted in compliance with the South African animal welfare legislation, the Good Clinical Practice guideline (Veterinary International Conference on Harmonization GL9) and the European Medicines Agency guidelines for testing and evaluation of the efficacy of anti-parasitic substances for treatment and prevention of tick and flea infestation in dogs and cats (EMEA/CVMP/005/2000-Rev.2). The studies employed a parallel group design, randomised and blinded. The dogs were ranked, within gender in descending order of individual body weight on Day -7. All dogs, identifiable by a microchip number, were individually housed in tick-proof kennels and observed daily throughout the study duration. Persons involved in the post-treatment assessments and observations were different from those that performed the treatments with the active ingredients in order to eliminate bias.

Both studies were conducted on two groups of eight dogs each. The dogs had not been treated with any ecto-parasiticide for 12 weeks prior to the start of the study. For the *B. canis* study, the dogs were tested sero-negative for *B. canis* by IFA and negative for *Babesia* DNA by PCR. A further 16 dogs were randomly allocated to one of two groups for the *Ehrlichia* study. They were admitted to the study after they were confirmed sero-negative for ehrlichiosis by IFA as well as PCR negative for *Ehrlichia* DNA.

The treatment consisted of 6.76 mg/kg fipronil and 50.48 mg/kg permethrin applied by parting the hair and applying the acaricide directly onto the skin along the midline of the neck. The total amount was divided into two fractions: one was applied between the shoulders and one at the base of the skull. Dogs were observed hourly for 4 h following treatment administration.

### Tick challenge on dogs

A laboratory-bred *Dermacentor reticulatus* tick strain, originating from France, naturally infected with *B. canis*, was used. Ticks from the above mentioned strain were infected with *B. canis* by acquisition feeding on a dog with confirmed acute babesiosis. Unfed adult ticks with a balanced gender ratio (50 % female: 50 % male) were used for the dog infestations. A sample of 50 *D. reticulatus* ticks taken from the batch of ticks to be used was confirmed positive by PCR analysis (rate ranging between 2 and 10 %). Each dog was infested on days 7, 14, 21 and 28 with 50 (±5) viable ticks applied directly onto the back of the dog. During this process dogs were restrained for 10 min in an infestation crate. Gloved fingers were used to facilitate the ticks through the dog’s hair coat in order to reach the skin. Any tick that was found dislodged during the first ten min was placed back onto the dog.

In the second study, a laboratory-bred *R. sanguineus* tick strain, originating from France and infected with *E. canis,* was used for the dog infestations. Ticks from the above mentioned strain were infected with *E. canis* by acquisition feeding on a dog with confirmed acute ehrlichiosis. A sample of 50 *R. sanguineus* ticks from the batch used for infestation was confirmed infected with *E. canis* with an infection rate of 13 % by PCR analysis. Fifty (± 5) unfed adult ticks of equal gender were released in the sleeping kennels of the dogs on days 7, 14, 21, 28, 35, 42, 49 and 56. Once a dog became infected with *E. canis* and was subsequently rescue-treated, adult *R. sanguineus* ticks from a pathogen-free batch were used for the artificial infestations (on Days 49 and 56) for a comparison of the acaricidal efficacy between groups. Additionally, the pens of the dogs were inspected daily from Day 14 onwards for engorged detached ticks. These ticks were collected and preserved in 70 % ethanol from each individual animal.

### Tick counts on dogs

In the *Babesia* study, *in situ* thumb counts were performed approximately 48 h after each tick challenge (Days 9, 16 and 23). Ticks were removed only 48 h after the last infestation (Day 30). In the *Ehrlichia* study, ticks were counted approximately 48 h post-application on the dogs without removing them (Days 9, 16, 23, 30, 37, 44, and 51). All ticks were removed on the final assessment day (Day 58). During the *in situ* thumb counts, sexes were not distinguished. The ticks counted and removed on Day 58 were categorized within sex (male/female) as free or attached and dead or alive following the recommendations recently updated by the WAAVP [12].

### Methods for calculating the acaricidal efficacy

Efficacy against ticks was calculated from the total count of live ticks counted on the dogs 48 h after each infestation, or removed. Efficacy calculations based on arithmetic and geometric means of the tick counts was calculated using Abbott’s formula: Efficacy (%) = 100 × (C – T) / C, whereby: C = Mean live tick count on the control group; T = Mean live tick count on the treated group. Statistical analysis were carried out using the chi-square test or Fisher’s exact test as applicable using software package SAS **®** version 9.3. The level of significance of the tests was set at 5 %.

### Monitoring of *Babesia* and *Ehrlichia* infections

#### *Babesia*

Scheduled clinical examinations were conducted on Days −7, 7, 14, 21, 28, 35, 42, 49 and 56. The clinical examination included general appearance, respiration rate, heart rate and body temperature. Additional examinations were conducted on all dogs displaying clinical signs associated with babesiosis, which included fever, depression, anorexia, lethargy, anaemia, haematuria and icterus. Blood smears were prepared from dogs displaying abnormally high body temperatures (>39.4 °C) and examined for *B. canis* infection in erythrocytes. Treatment for babesiosis consisted of 1 ml/20 kg body weight diminazene followed by 1.2 ml/kg imidocarb dipropionate 24 h later.

Blood was collected for PCR analysis from all dogs prior to the start of the study and on Days 14, 21, 28, 35, 42 and 56 and on any dog at the time of diagnosis with babesiosis prior to rescue treatment. Blood was also collected for serology on the same days as for PCR analyses, and additionally on Day 7 prior to the tick challenge. EDTA blood samples collected for PCR analysis were collected in EDTA tubes and total genomic DNA extracted using a commercial kit. A fragment of approximately 300 bp from the 18S internal transcribed spacer-1 gene of *Babesia* was PCR amplified using methods originally published by Duarte et al. [24] and subsequently modified by Beugnet et al. [19]. Positive, negative, no template as well as internal amplification controls were included in each run.

For serology, serum samples were examined for the presence of *B. canis*-specific antibodies using IFA according to the instructions of the manufacturer (MegaCor Diagnostik, Austria).

#### *Ehrlichia*

Infection with *E. canis* was monitored by rectal temperature records, clinical examinations and platelet counts, as well as by testing blood samples by PCR and IFA. Dogs displaying clinical signs usually based on an elevated body temperature >39.4 °C for two consecutive days received appropriate concomitant treatment with doxycycline and dexamethasone. EDTA blood samples were collected for PCR from all dogs prior to the first infestation and on Days 21, 28, 35, 42, 49, 56, 63, 70, 77 and 84, as well as any dogs that developed fever.

For PCR analysis of *E.canis*, a specific primer set was used for amplification of a fragment of the dsb gene (ECAdsbF: 5′- GCAAGTGCGGGCAGAGAATGAAG-3′; ECAdsbR: 5′- GTATCCCCTACTATGATAGCAGGAGTGC-3′). The amplified product was subjected to agarose gel electrophoresis for confirmation. Up to 400 ng isolated DNA served as template for PCR amplification of the target region in a 20 μl reaction volume using Phire HotStart II DNA polymerase. A PCR product of approximately 500 bp confirmed the presence of the *E. canis* dsb target region in the sample. Positive, negative, no template as well as internal amplification controls were included in each run.

For serology, serum was recovered and frozen at −20 °C until assayed for *E. canis* antibodies using an IFA assay for the detection of specific *E. canis* antibodies using a commercial test kit (MegaCor Diagnostik, Austria). An additional serum sample was collected three weeks after the last scheduled serum collection on Day 84, to confirm the results for dog 4FA 06A that had not sero-converted by the end of study.

### Methods for calculating the *Babesia/Ehrlichia* blocking efficacy

An efficacy failure (successfully infected with *Babesia*) was defined as a dog in the treatment group that tested serologically positive for *B. canis* antibodies or positive for *B. canis* DNA by PCR analysis. An efficacy failure (successfully infected with *Ehrlichia*) was defined as a dog in the treatment group that tested serologically positive for *E. canis* antibodies or tested positive for *E. canis* DNA by PCR analysis. Any treated dog that met either one of the above criteria was considered infected. Percentage blocking efficacy for the treatment group was calculated as follows: Efficacy (%) = 100 × (T_c_ − T_t_)/T_c_, whereby T_c_ = Total number of infected dogs in the negative control group, and, T_t_ = Total number of infected dogs in the treatment group.

As proposed recently by Navarro et al. 2015 [25], the percentage of protection may also be calculated in comparison to the number of infective challenges, and not in relation to the number of infected dogs in the control groups.

Protection (%) = 100 (IcC − IcT)/IcC whereby IcC is the number of infective tick challenges conducted in the control group that lead to positive infection and IcT the number of infective tick challenges in the treated dogs that lead to infection. This % of protection provides a better view of the risk reduction provided by the treatment to dogs that will face infected tick challenges.

## Results

In general, clinical signs, fever and reduced platelet counts, observed in dogs enrolled in the studies could be linked to the tick-transmitted *Babesia* or *Ehrlichia i*nfections, and there were no adverse reactions noted in response to the treatment.

### Efficacy on ticks

The acaridical efficacy of Frontline Tri-Act®/Frontect® against *D. reticulatus* and *R. sanguineus* ticks is summarised in Table 1 and Table 2, respectively. The efficacy against *D. reticulatus* ticks was 98.3 % on Day 9 and increased to 100 % on Day 16 (Table 1). Any dog that tested positive for *B. canis* parasites in stained blood smears was not challenged any further with infected *Dermacentor* ticks. As a result, meaningful statistical comparison was limited to Days 9 and 16 (Table 1).

**Table 1:** Acaricidal efficacy based on geometric and arithmetic means against *Dermacentor reticulatus* ticks

Day	Geometric means			Arithmetic means		
	Mean	Mean (Efficacy %)	P-value	Mean	Mean (Efficacy %)	P-value
	Control group	Treated group		Control group	Treated group	
Day 9	23.1	0.4 (98.3 %)	<.0001	24.5		0.9 (96.4 %)
Day 16	30.6	0.0 (100.0 %)	<.0001	32.7		0.0 (100.0 %)
Day 23	NA	0.5				1.4
Day 30	NA	0.3				0.6

P-value: One-way ANOVA test

Dogs were treated once on day 0

*NA* Not Applicable, dogs removed from the study after babesiosis diagnosis

**Table 2:** Acaricidal efficacy bases on geometric and arithmetic means against *Rhipicephalus sanguineus* ticks

Day	Geometric means			Arithmetic means		
	Mean	Mean (Efficacy %)	P-value	Mean	Mean (Efficacy %)	P-value
	Control group	Treated group		Control group	Treated group	
Day 9	8.9	0.1 (99.0 %)	<.0001	10.4	0.1 (98.8 %)	0.0004
Day 16	18.0	0.0 (100.0 %)	<.0001	18.5	0.0 (100.0 %)	<.0001
Day 23	18.1	0.0 (100.0 %)	<.0001	18.4	0.0 (100.0 %)	<.0001
Day 30	18.4	0.0 (100.0 %)	<.0001	19.1	0.0 (100.0 %)	<.0001
Day 37	18.5	0.0 (100.0 %)	<.0001	19.0	0.0 (100.0 %)	<.0001
Day 44	20.9	0.0 (100.0 %)	<.0001	25.3	0.0 (100.0 %)	0.0038
Day 51	5.6	0.0 (100.0 %)	<.0001	7.4	0.0 (100.0 %)	0.0038
Day 58	7.5	0.0 (100.0 %)	<.0001	10.0	0.0 (100.0 %)	0.0065

P-value: One-way ANOVA test

Dogs were treated on day 0 and again on day 28

Live *R. sanguineus* ticks were only found on Day 9 with a corresponding efficacy of 99.0 % for the treatment group (Table 2). The control group carried statistically (*p* < 0.05) more ticks compared to the treated group on all assessment days with a group average of 5.6 to 20.9 (Table 2). After Day 9, no more live ticks could be found on the treated dogs on any of the assessment days (i.e. 100 % efficacy).

#### *Babesia canis* blocking efficacy

The infection rate of ticks used for infestation on day 7 was 2 %, whereas those used on Days 21 and 28 carried an infection rate of 10 and 8 %, respectively.

Blood smears were prepared and examined for the presence of *B. canis* for all dogs from Day 14 onwards when pyrexia (>39.4 °C) was present. *B. canis* was observed in blood smears of all control dogs on at least one occasion (Table 3). By Day 28, all the dogs in the control group were positive for *Babesia*, and therefore for those animals tick challenges were discontinued. For all treated dogs, tick challenges were continued up to Day 28, except for dog B2A 234 from the treatment group, confirmed positive on Day 22.

**Table 3:** Rectal temperature records and detection of *Babesia canis* in blood smears from dogs challenged with infected *Dermacentor* ticks

Group	Animal ID	Body temp range (°C)		Blood smear preparation and examination day											
				14	15	16	17	20	21	22	28	35	42	49	56
		Min	Max												
Control	4F3 1A0	37.7	40.3	-	POS	-	-	-	-	-	-	-	-	-	-
	B25 46D	37.9	39.1	POS	-	-	-	-	-	-	-	-	-	-	-
	B29 74B	38.2	39.7	ND	POS	-	-	-	ND	-	ND	ND	ND	ND	ND
	B2C 449	37.8	38.8	-	-	-	-	-	POS	-	-	-	-	-	-
	CC0 CE3	37.6	39.6	ND	ND	POS	-	-	ND	-	ND	ND	ND	ND	ND
	CC2 25E	37.6	39.2	-	-	-	POS	-	-	-	-	-	-	-	-
	CC2 726	37.5	40.1	ND	POS	-	-	-	-	-	-	-	-	-	-
	E9E 126	37.9	39.8	POS	-	ND	-	-	ND	ND	ND	ND	ND	ND	ND
Treated	B2A 234	37.7	40.5	ND	-	-		ND	ND	POS	ND	ND	ND	ND	ND
	B2B 68D	38.4	39.2	-	-	-	-	-	-	-	-	-	-	-	-
	DF6 4EF	37.8	38.8	-	-	-	-	-	-	-	-	-	-	-	-
	DF6 576	38.0	39.0	ND	-	-	-	-	-	-	ND	ND	-	ND	ND
	DF6 725	37.4	39.1	ND	-	-	-	-	ND	-	ND	ND	-	-	ND
	DF7 D38	37.6	38.4	-	-	-	-	-	-	-	-	-	-	-	-
	E15 564	37.5	38.4	-	-	-	-	-	-	-	-	-	-	-	-
	E46 966	37.3	39.2	ND	-	-	-	-	-	-	-	-	-	ND	-

*ND* not detected, *POS* positive, - = no blood smear prepared

Blood smear examination was followed up by PCR and IFA analysis. All eight untreated dogs were confirmed positive by PCR on the same day as their positive blood smear (Table 4). By Day 28 all untreated dogs had sero-converted and displayed specific *B. canis* antibodies (Table 5). One of the dogs (B2A 234) in the treated group was found positive for babesiosis based on blood smear examination (Day 22) (Table 3), PCR (Day 21) (Table 4) and IFA (Day 42) (Table 5). Overall the effectiveness of Frontline Tri-Act®/Frontect® in reducing *Babesia* transmission was 87.5 % over the challenge period of 28 days compared to control dogs (P-value: 0.0014). When calculating the protection conferred against infective tick challenges, the percentage of protection was 94.3 % ([8/15 − 1/31)/8/15] = 1 infection in 31 infective challenges in treated dogs compared to eight infections in 15 challenges in control dogs).

**Table 4:** Detection of *Babesia canis* DNA using a PCR assay in dogs challenged with infected *Dermacentor* ticks

	Animal ID	DAY										
		14	15	16	17	21	22	28	35	42	49	56
Control	4F3 1A0	-	POS	-	-	ND	-	-	-	-	-	-
	B25 46D	POS	-	-	-	ND	-	-	-	-	-	-
	B29 74B	-	POS	-	-	ND	-	-	-	-	-	-
	B2C 449	-	-	-	-	POS	-	-	-	-	-	-
	CC0 CE3	-	-	POS	-	ND	-	-	-	-	-	-
	CC2 25E	-	-	-	POS	ND	-	-	-	-	-	-
	CC2 726	-	POS	-	-	ND	-	-	-	-	-	-
	E9E 126	POS	-	-	-	ND	-	-	-	-	-	-
Treated	B2A 234	-	-	-	-	POS	POS	-	-	-	-	-
	B2B 68D	-	-	-	-	ND	-	ND	ND	ND	ND	ND
	DF6 4EF	-	-	-	-	ND	-	ND	ND	ND	ND	ND
	DF6 576	-	-	-	-	ND	-	ND	ND	ND	ND	ND
	DF6 725	-	-	-	-	ND	-	ND	ND	ND	ND	ND
	DF7 D38	-	-	-	-	ND	-	ND	ND	ND	ND	ND
	E15 564	-	-	-	-	ND	-	ND	ND	ND	ND	ND
	E46 966	-	-	-	-	ND	-	ND	ND	ND	ND	ND

*POS* Positive, *ND* Not detected; - = No sample tested

**Table 5:** Detection of *Babesia canis* antibodies by Indirect Fluorescent Antibody assay in dogs challenged with infected *Dermacentor* ticks

	Animal ID	DAY							
		Pre -infestation Day - 7	7	21	28	35	42	49	56
Control	4F3 1A0	NEG	NEG	POS	-	-	POS	POS	POS
	B25 46D	NEG	NEG	POS	-	-	POS	POS	POS
	B29 74B	NEG	NEG	NEG	POS	-	POS	POS	POS
	B2C 449	NEG	NEG	NEG	POS	-	POS	POS	POS
	CC0 CE3	NEG	NEG	NEG	POS	-	POS	POS	POS
	CC2 25E	NEG	NEG	POS	-	-	POS	POS	POS
	CC2 726	NEG	NEG	POS	-	-	POS	POS	POS
	E9E 126	NEG	NEG	NEG	POS	-	POS	POS	POS
Treated	B2A 234	NEG	NEG	NEG	NEG	NEG	POS	POS	POS
	B2B 68D	NEG	NEG	NEG	NEG	NEG	NEG	NEG	NEG
	DF6 4EF	NEG	NEG	NEG	NEG	NEG	NEG	NEG	NEG
	DF6 576	NEG	NEG	NEG	NEG	NEG	NEG	NEG	NEG
	DF6 725	NEG	NEG	NEG	NEG	NEG	NEG	NEG	NEG
	DF7 D38	NEG	NEG	NEG	NEG	NEG	NEG	NEG	NEG
	E15 564	NEG	NEG	NEG	NEG	NEG	NEG	NEG	NEG
	E46 966	NEG	NEG	NEG	NEG	NEG	NEG	NEG	NEG

*POS* Positive, *NEG* Negative; - = No sample tested

#### *Ehrlichia canis* blocking efficacy

Fifty adult *R. sanguineus* ticks were taken from the batch of ticks used for challenging the dogs and confirmed PCR positive (13 %). In both groups, four dogs were observed with elevated body temperatures (>39.4 °C) (Table 6). Reduced platelet counts (< 200 × 10^9^/l) were observed in five untreated dogs, but also in four of the treated dogs (Table 6). *Ehrlichia canis* infection was confirmed by PCR in all untreated dogs (Table 7) and they also all seroconverted (Table 8). *Ehrlichia* DNA was detected in two treated dogs (4DA C4C and 4FA 06A on Day 70 and Day 77, respectively) (Table 7). However, only one of the PCR positive dogs in the treated group was confirmed by IFA (Table 6). Additional serum samples collected from dog 4FA 06A after Day 84 were also sero-negative. Overall, Frontline Tri-Act®/Frontect® effectively reduced transmission of *E.canis* to dogs by 75 % over the challenge period of 56 days compared to control dogs (P-value: 0.0070). When calculating the protection conferred against infective tick challenges, the percentage of protection was 85.15 % (two infections based on PCR in 64 infective challenges compared to eight infections in 38 challenges in control dogs).

**Table 6:** Rectal temperature records and platelet counts in dogs challenged by *Ehrlichia canis*-infected *Rhipicephalus* ticks

	Animal ID	Body temp range (°C)		Platelet count and examination Day											
		Min	Max	−6	21	28	35	36	42	49	56	63	70	77	84
Control	CC5 CDA	37.5	39.7	285	275	292	284	-	221	-	-	-	-	-	-
	CD6 3F9	37.5	38.8	224	234	218	**189**	-	-	-	-	-	-	-	-
	EA1 FF0	37.7	39.8	245	**171**	-	246	-	-	-	-	-	-	-	-
	4F1 4AF	38.1	40.1	494	415	519	449	-	459	383	388	274	-	-	-
	4F0 57A	37.8	39.4	253	282	279	**197**	-	-	-	-	-	-	-	-
	4F6 87C	38.2	39.8	333	327	**194**	**179**	-	-	-	-	-	-	-	-
	286 FFE	38.4	39.4	485	478	512	505	-	495	406	**143**	-	-	-	-
	964 441	37.8	39.1	377	349	359	353	-	269	-	-	-	-	-	-
Treated	CBD 700	37.3	38.6	213	238	216	-	209	221	210	**193**	208	213	185	193
	B2C 3F0	38.2	39.6	259	**192**	**192**	**169**	**-**	**153**	**181**	**198**	216	170	204	180
	28A 3C2	38.1	39.8	363	343	307	380	-	370	348	351	376	349	325	308
	E18 F40	38.0	39.2	480	228	304	250	-	299	280	280	288	312	275	277
	4F0 890	38.2	39.6	347	367	332	330	-	372	341	204	378	349	339	332
	4DA C4C	38.2	39.2	425	385	402	360	-	299	320	291	323	**163**	-	-
	DF7 4DB	37.7	39.1	248	273	268	255	-	244	248	259	314	252	265	244
	4FA 06A	37.6	39.7	249	281	256	250	-	244	269	235	**181**	228	202	-

Normal range for platelet count is between 200 × 10^9^/l and 500 × 10^9^/l (below normal range indicated in bold)

- = no platelets were counted

**Table 7:** Detection of *Ehrlichia canis* DNA using a PCR assay in dogs challenged with infected *Rhipicephalus* ticks

	Animal ID	Day										
		−6	21	28	35	42	49	56	63	70	77	84
Control	CC5 CDA	ND	ND	ND	ND	POS	-	-	-	-	-	-
	CD6 3F9	ND	ND	ND	POS	-	-	-	-	-	-	-
	EA1 FF0	ND	POS	-	ND	**-**	**-**	**-**	**-**	**-**	**-**	**-**
	4F1 4AF	ND	ND	ND	ND	ND	ND	ND	POS	-	-	-
	4F0 57A	ND	ND	ND	POS	-	-	-	-	-	-	-
	4F6 87C	ND	ND	POS	POS	-	-	-	-	-	-	-
	286 FFE	ND	ND	ND	ND	ND	ND	POS	-	-	-	-
	964 441	ND	ND	ND	ND	POS	-	-	-	-	-	-
Treated	CBD 700	ND	ND	ND	ND	ND	ND	ND	ND	ND	ND	ND
	B2C 3 F0	ND	ND	ND	ND	ND	ND	ND	ND	ND	ND	ND
	28A 3C2	ND	ND	ND	ND	ND	ND	ND	ND	ND	ND	ND
	E18 F40	ND	ND	ND	ND	ND	ND	ND	ND	ND	ND	ND
	4 F0 890	ND	ND	ND	ND	ND	ND	ND	ND	ND	ND	ND
	4DA C4C	ND	ND	ND	ND	ND	ND	ND	ND	POS	-	-
	DF7 4DB	ND	ND	ND	ND	ND	ND	ND	ND	ND	ND	ND
	4FA 06A	ND	ND	ND	ND	ND	ND	ND	ND	ND	POS	-

*POS* Positive, *ND* Not detected; - = Not tested

**Table 8:** Detection of *Ehrlichia canis* antibodies by Indirect Fluorescent Antibody assay in dogs challenged with infected *Rhipicephalus* ticks

	Animal ID	DAY										
		7	21	28	35	42	49	56	63	70	77	84
Control	CC5 CDA	NEG	NEG	NEG	NEG	POS	POS	POS	POS	POS	POS	POS
	CD6 3F9	NEG	NEG	NEG	POS	POS	POS	POS	POS	POS	POS	POS
	EA1 FF0	NEG	POS	POS	POS	POS	POS	POS	POS	POS	POS	POS
	4F1 4AF	NEG	NEG	NEG	NEG	NEG	NEG	NEG	NEG	POS	POS	POS
	4F0 57A	NEG	NEG	NEG	POS	POS	POS	POS	POS	POS	POS	POS
	4F6 87C	NEG	NEG	NEG	POS	POS	POS	POS	POS	POS	POS	POS
	286 FFE	NEG	NEG	NEG	NEG	NEG	NEG	POS	POS	POS	POS	POS
	964 441	NEG	NEG	NEG	NEG	POS	POS	POS	POS	POS	POS	POS
Treated	CBD 700	NEG	NEG	NEG	NEG	NEG	NEG	NEG	NEG	NEG	NEG	NEG
	B2C 3F0	NEG	NEG	NEG	NEG	NEG	NEG	NEG	NEG	NEG	NEG	NEG
	28A 3C2	NEG	NEG	NEG	NEG	NEG	NEG	NEG	NEG	NEG	NEG	NEG
	E18 F40	NEG	NEG	NEG	NEG	NEG	NEG	NEG	NEG	NEG	NEG	NEG
	4F0 890	NEG	NEG	NEG	NEG	NEG	NEG	NEG	NEG	NEG	NEG	NEG
	4DA C4C	NEG	NEG	NEG	NEG	NEG	NEG	NEG	NEG	POS	POS	POS
	DF7 4DB	NEG	NEG	NEG	NEG	NEG	NEG	NEG	NEG	NEG	NEG	NEG
	4FA 06A	NEG	NEG	NEG	NEG	NEG	NEG	NEG	NEG	NEG	NEG	NEG

*POS* Positive, *NEG* Negative

## Discussion

### Acaricidal efficacy

Topical administration of a combination of fipronil and permethrin onto eight dogs enrolled in each of the clinical studies included in this paper did not induce any adverse reactions. Any clinical signs observed were linked to either *B. canis* infection or to *E.canis* infection.

The advantage of combining 6.76 % fipronil and 50.48 % permethrin is their different mode of action. Permethrin has a pronounced repellency effect related to irritant effect by contact, and then is followed by a killing effect. Fipronil induces a progressive onset of tick mortality [23]. “Synergistic” effects, or at least additive effects, by combining both topical compounds into a single formulation, as discovered for the combination of fipronil and amitraz [26], have not been reported but are probable.

### Speed of transmission

Pathogen transmission depends on the duration of attachment required by ticks to transmit specific pathogens such as *B. canis* and *E. canis.* In general, protozoan *Babesia* parasites require several days (36 to 72 h) for their sporoblasts to mature into infective sporozoites within the tick’s salivary glands before they can be transmitted [27]. Bacterial pathogens, such as *Anaplasma phagocytophilum,* require 24 to 36 h to be transmitted by nymphal *Ixodes scapularis* ticks. [28, 29]. In a recent study, the time that an infected *R. sanguineus* tick had to be attached before it could transmit *E. canis* was determined in vivo as well as *in vitro* [30]. The study revealed that transmission of *E. canis* starts within a few hours (3 h on dogs and 8 h on artificial membranes), an interval considerably shorter than presumed previously. These findings highlight the need for further research concerning the actual speed of transmission of tick-borne pathogens.

As a result, the preventive efficacy of ecto-parasiticides with respect to blocking pathogen transmission has become an important issue in advice from veterinarians towards pet owners.

### Transmission blocking efficacy

The blocking capacity of various acaricidal compounds against infected *D. reticulatus* ticks has been evaluated in a series of clinical laboratory studies [10, 16, 19, 20, 25]. For instance, the ability to block transmission of *B. canis* by *D. reticulatus* to dogs was recently demonstrated for afoxolaner [19] as well as for fluralaner [20]. Moreover, blocking of the transmission of *E. canis* has also been evaluated in a number of similar studies with other acaricidal molecules [11, 14, 15].

Recently, prevention of *B. canis* by a fixed combination of permethrin and fipronil (Effitix®) using the blocking model with infected *D.reticulatus* ticks was reported [25].

When calculating the protection conferred against *Babesia*-infected tick challenges, the percentage of protection was 93.4 % (one infection in 31 infective challenges versus eight infective challenges in 15 in control dogs). Likewise, the percentage of protection against *Ehrlichia*-infective tick challenges was 85.15 % (two infections based on PCR in 64 infective challenges compared to eight infections in 38 challenges in control dogs). This approach allowed for a more realistic estimate of the repeated tick challenge of the dogs without the need for additional dogs.

Another interesting issue is the definition of an efficacy failure or success when executing blocking models. Per definition transmission blocking implies the prevention of any babesial sporozoites or ehrlichial organisms from passing from the tick vector to the host. A dog that has sero-converted and/or tested positive for *B. canis* or *E. canis* DNA by PCR is therefore regarded as an efficacy failure, irrespective of any clinical disease manifestation [13]. However, it can also be argued that successful transmission of a pathogen should result in clinical disease. It is possible that a dog that sero-converted or tested positive by PCR did not develop any clinical signs due to insufficient challenge. An acaricidal product can potentially disrupt the feeding process sufficiently to prevent transmission of a viable infection load of either *B. canis* or *E. canis*. In that case, prevention of disease transmission should be calculated in regard to the number of dogs developing clinical signs and confirmed by either PCR or serology. Nevertheless, it is the opinion of the authors that the definition of a successfully infected *B. canis* dog was used by Navarro et al*.* [25], stating that infected dogs must be PCR positive and seropositive is not acceptable. We consider that in terms of infection, PCR is a proof that the pathogen has been inoculated, as well as seropositivity. Therefore, it is our opinion that one or the other should be regarded as an efficacy failure [10].

Another improvement of the protocol for these models includes the way ticks are brought into contact with the dogs. *R. sanguineus* ticks were placed in the dog’s kennel [11], whereas *D. reticulatus* was placed directly onto the dogs. This is considered in line with differences in host seeking behaviour of both tick species. Moreover, re-infestation with non-infected ticks after a dog has become positive was introduced in the protocol of the *Ehrlichia* study, which resulted in a meaningful statistical comparison between groups throughout the study (Table 2).

In the *Ehrlichia* study, dogs were monitored for thrombocytopenia by determining platelet counts in non-infected dogs ranging between 200 × 10^9^/l and 500 × 10^9^/l. Platelet counts below 200 × 10^9^/l were detected in both groups as different time points and did not correlate with an elevated body temperature (Table 6). In fact, in the control group there were only two dogs (EA1 FF0 and 4F6 87C) with fever and low platelet counts, whereas in the treated group there were also two dogs (B2C 3F0 and 4FA 06A) with fever and lower platelet values (Table 6). Clearly, sero-conversion and PCR positivity are better criteria than platelet counts. Nevertheless, thrombocytopenia is a characteristic of monocytic ehrlichiosis, but differs between individual dogs and between time points collected from the same dogs [31].

In these studies, dogs were challenged with either *D. reticulatus* ticks with *Babesia* infection between 2 and 10 % with *B. canis* or with *R. sanguineus* ticks carrying an *Ehrlichia* infection rate of around 13 %. Tick infection rates in field collections vary between publications and depend upon which publication is cited. However, the challenge load in both models appears fairly realistic when compared to an *E. canis* incidence risk in dogs in southern Europe of 11 % [32] and with an infection rate in *D. reticulatus* field ticks of 1.64 % recently determined in the Netherlands [33].

## Conclusions

The findings presented here demonstrate that a combination of 6.76 % *w/v* fipronil and 50.48 % *w/v* permethrin was able to reduce transmission of *B. canis* as well as *E. canis* to dogs.
